# Hospitals for Measles-Infected Children

**Published:** 1897-11-27

**Authors:** 


### HOSPITALS FOR MEASLES-INFECTED CHILDREN.

It is not often that we are found advocating the establishment of new hospitals, and especially of hospitals for the treatment of any special group of disorders, but it is being borne in upon us with added force every day that there is a great gap in the hospital resources of London in this respect—that practically no accommodation exists for treatment of those complications which so commonly occur in the course of measles, and which give to that disease its high fatality. We hear much of scarlet fever and diphtheria, and the latter of these diseases is generally admitted to have become a pest and a danger of the first magnitude to the inhabitants of London. Yet both scarlet fever and diphtheria together do not carry off so many victims as are claimed by measles alone. It must also be understood that, far more than is the case with either scarlet fever or diphtheria, these deaths from measles are preventable. Measles by itself is far from being a fatal disease; it is fatal chiefly by its complications, and these complications are to a very considerable extent the result of the conditions amid which the patients live. Yet, practically, we have no means of removing these little sufferers to places where they might be properly tended and brought back to health. They cannot be taken into the metropolitan fever hospitals, and they can only be admitted to children's hospitals and to such general hospitals as contain children at the risk of an outbreak of the disease among the other patients. So long as the infection lasts, they are outside the pale in regard to hospital treatment. Last year 3,697 persons died from measles in London alone. No record is kept of the number of cases which have occurred, but if so many actually died it must have been very great; and we can but imagine how vast is the number of sick children who are suffering, in one way and another, from the complications and sequelæ of the disease. The notification of measles and its treatment in rate-supported hospitals is another question, which hinges largely on the view taken as to the possibility of isolating so ubiquitous a disease. Of this we now say nothing; but we do urge that, with our modern knowledge regarding the cause of death in many cases of measles, the question of the hospital treatment of the complications and sequelæ of the disease becomes a matter of considerable urgency.

